# Biomechanical Properties in Different Types of Thin Corneas in Menoufia Population

**DOI:** 10.1155/2021/6613143

**Published:** 2021-01-04

**Authors:** Adel Galal Zaky, Amin Faisal Ellakwa, Ahmed Ibrahim Basiony

**Affiliations:** Ophthalmology Department, Faculty of Medicine, Menoufia University, Shebin El Kom, Egypt

## Abstract

**Background:**

To evaluate and compare corneal hysteresis (CH) and corneal resistance factor (CRF) in normal thin (NT) healthy corneas with central corneal thickness (CCT) of 470–500 *μ*m with matched thickness in keratoconus suspect (KCS) and keratoconus (KC) eyes.

**Methods:**

A total of 103 eyes in three groups were included prospectively: NT, KCS, and KC groups based on clinical examination and Pentacam findings. Corneal hysteresis (CH) and corneal resistance factor (CRF) were measured using the ocular response analyzer (ORA). CCT, CH, and CRF were compared between the three groups and statistically analyzed by variance tests.

**Results:**

The three groups consisted of 44 NT, 26 KCS, and 33 KC. The mean CH measured was 8.689 ± 1.775, 9.051 ± 1.1190, and 8.129 ± 0.8539 mmHg in NT, KCS, and KC eyes, respectively. The mean CRF was 8.441 ± 1.663, 8.337 ± 1.114, and 7.2422 ± 1.3110 mmHg in NT, KCS, and KC eyes, respectively. Within the range of central corneal thickness (470–500 *μ*m), only mean CRF was statistically significantly different between the NT and KC (*P* < 0.05); there was no statistically significant difference between NT and KCS, nor was the mean CH between each group (*P* > 0.05).

**Conclusions:**

CRF only can be helpful in differentiating KC from NT eyes; KCS could not be predicted with either corneal biomechanical metrics. There was no benefit from CH in differentiating between the three study groups.

## 1. Background

Central corneal thickness (CCT) is a biometric factor [1] with a wide range of variability in healthy eyes, the cause of which is believed to result from different amounts of collagen fibrils and interfibrillar substance in the corneal stromal matrix [2]. It is a measure of tissue mass and represents an indicator of corneal rigidity. Also, CCT changes among ethnic groups and shows strong heritability among families [3].

The development of a test for reliable assessment of corneal rigidity and its response to excimer laser ablation was a vital point in the development of refractive surgery. This was a challenging issue until 2005, when the ocular response analyzer (ORA) appeared in the market with its uses in ophthalmology medicine [4].

The ORA has an infrared electrooptical system that monitors corneal deformations. It delivers a precisely metered collimated air pulse to the eye. The cornea suffers an inward movement, passing a first applanation state before assuming a concave shape. The air pressure progressively declines after this first applanation and the cornea passes through a second applanation state while returning to its normal convex curvature. The test plots a waveform that contains two peaks, corresponding to the inward and outward applanation moments [4].

Using this bidirectional applanation measurement, the ORA is able to present the four original parameters. Corneal hysteresis (CH) is the difference between these two pressure values, which represents the corneal viscoelastic damping. The mean of these two pressures is the Goldmann-correlated IOP (IOPg). The corneal-compensated IOP (IOPcc) is a pressure measurement that uses the CH to determine an IOP value that is less affected by corneal properties, such as CCT. Corneal resistance factor (CRF) is calculated using a proprietary algorithm and represents overall cornea resistance [5–7].

At present, CXL might be the first choice therapy to halt the progression of the early stages of corneal ectasia, showing good long-term visual results and few complications. Regarding the therapeutic benefit of CXL in stabilizing corneal ectasia progression, the early diagnosis of keratoconus and secondary corneal ectasia are mandatory. The target of the treatment is to increase the mechanical strength of the cornea halting the progression of keratoconus, avoiding or delaying recourse to keratoplasty [8, 9].

In the present study, we investigated the corneal biomechanical metrics in healthy eyes (NT) with CCT of 470 to 500 *μ*m and compared them with thickness matched keratoconus (KC) keratoconus suspect (KCS) cases.

## 2. Methods

This cross-sectional nonrandomized study was performed from December 2017 to November 2018 after receiving the approval of institutional ethical committee of Faculty of Medicine, Menoufia University, Egypt; all patients received a thorough explanation of the study design and aims; the study was conducted in compliance with informed consent regulations and family consent for subjects under 18 years. Patients were selected from ophthalmology outpatient clinics at Ophthalmology Department of Menoufia University Hospitals and Tiba Eye Center, Menoufia, Egypt.

In the current study, we have enrolled all subjects with central corneal thickness (CCT) measured at the thinnest location by Pentacam between 470 and 500 *μ*m, with the age ranging from 17 to 37 years. Keratoconus suspect was defined as thin corneas (470–500 *μ*m) with no clinical signs of keratoconus, steep keratometric reading greater than 47.0 diopters, minor topographic asymmetry (inferior-superior difference ≥1.5 D, superior-inferior difference ≥2.5 D), or borderline Belin Ambrosio display (BAD), whereas keratoconus group was defined as any grade of topographic keratoconus (according to Pentacam classification) with CCT within the range selected in the study (470–500 *μ*m).

Patients were divided into three groups, with their CCT between 470 and 500 *μ*m in pachymetric map of Pentacam: group NT: normal thin corneas with normal Pentacam; group KCS: corneas with suspicious Pentacam; group KC: corneas with keratoconus pattern in Pentacam.

All patients with previous ocular surgery, corneal scars or opacities, chronic use of topical medications, systemic collagen diseases, and previous history of corneal ulcers were rolled out from the study.

Each subject had a comprehensive ophthalmologic examination, including a review of their medical history, corrected distance visual acuity, slit lamp biomicroscopy, and fundus examination. Pentacam topography (oculus Pentacam, Optikgerate GmbH, Wetzlar, Germany) and every patient was subjected to ORA (ocular response analyzer, Reichert, Walden Ave, NY, USA) to measure corneal biomechanical parameters: corneal hysteresis (CH), corneal resistance factor (CRF), Goldmann-correlated pressure (IOPg), and corneal-compensated intraocular pressure (IOPcc).

**Figure 1:**
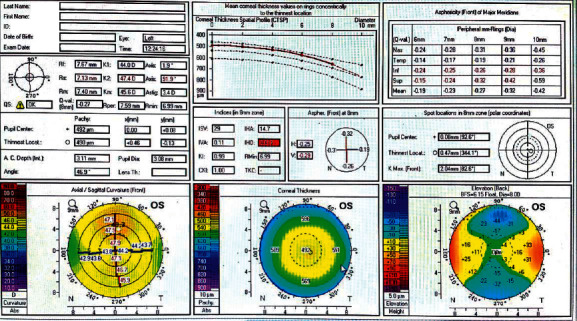
Pentacam of the left eye of a patient in group 1 (normal).

**Figure 2:**
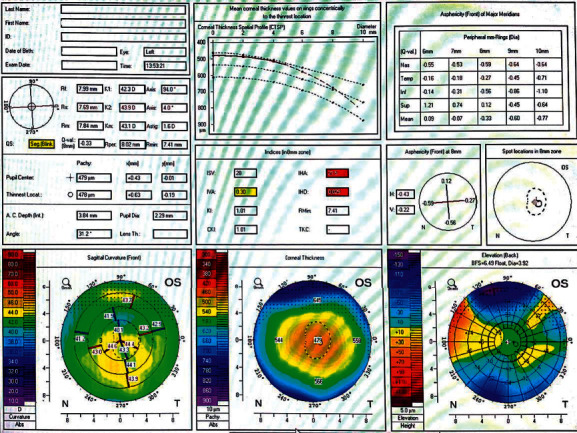
Pentacam of the left eye of a patient in group 2 (keratoconus suspect).

**Figure 3:**
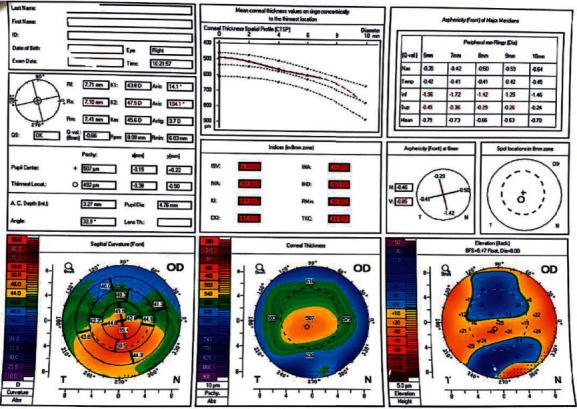
Pentacam of the right eye of a patient in group 3 (keratoconus).

Figures 1–3 represent Pentacam of study group 1 (normal thin cornea), group 2 (keratoconus suspect), and group 3 (keratoconus), respectively.

The ORA is a noncontact device with automated eye centration alignment. Subjects were seated on the examination chair and instructed to place their foreheads on the headrest of the ORA device and were instructed about a noncontact probe that would move toward the eye and emit a gentle puff of air. They were asked to fix on a blinking red light in the machine. Thereafter, the ORA was activated, and the air puff was emitted onto the center of the cornea. Only the reliable ORA readings with good score were obtained and stored. Two consecutive ORA measurements were made and the best waveform score from each patient was included in the analysis of the study.

The manufacturer defined good-quality readings as both force-in and force-out applanation signal peaks on the ORA waveform being symmetrical in height. The ORA displayed a graphic representation of the corneal response after each measurement.

**Figure 4:**
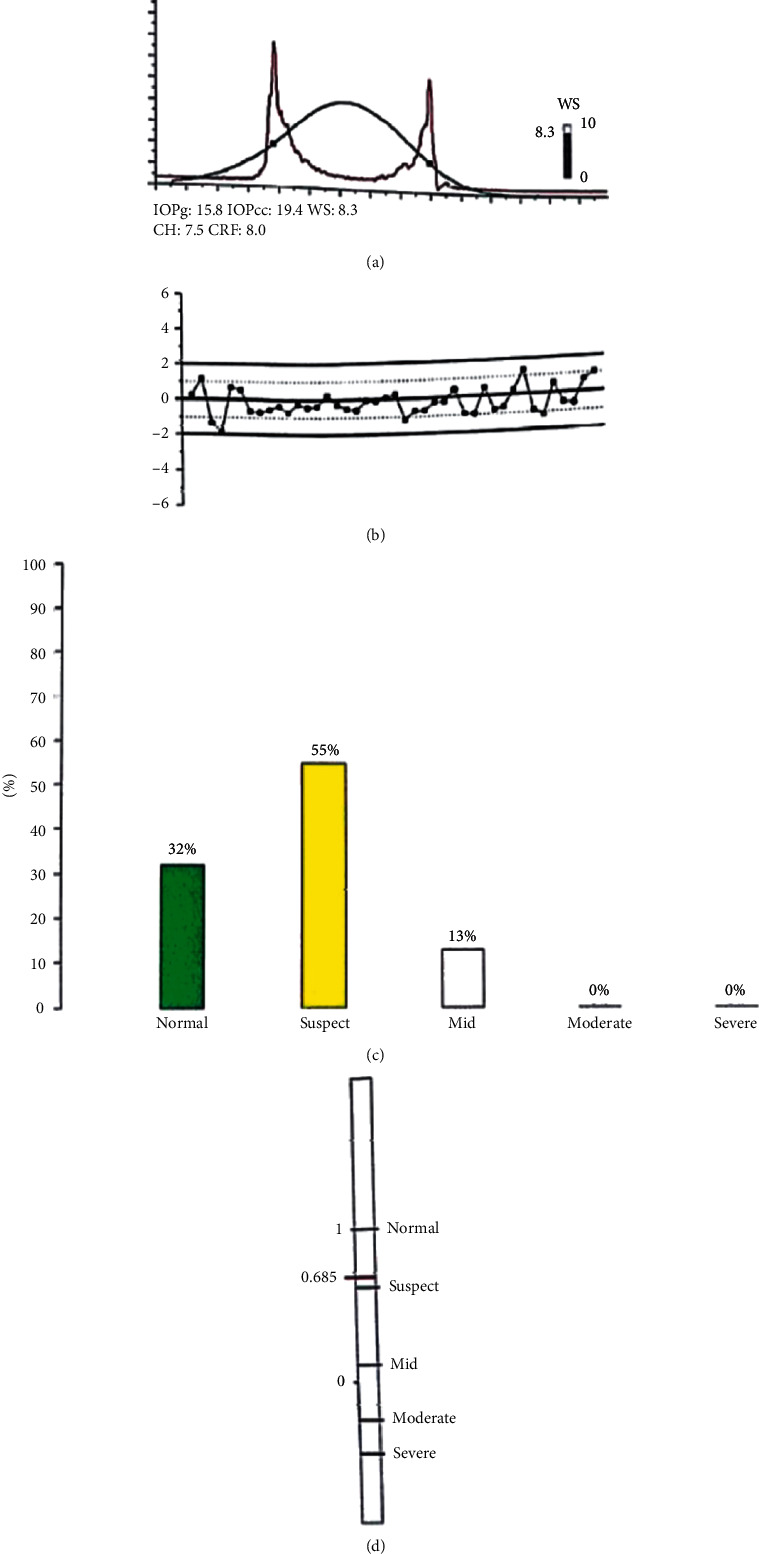
ORA signal of the left eye of a patient in group 1 (normal). (a) Waveform and measurement values. (b) Waveform parameters deviation from mean. (c) Keratoconus match probabilities. (d) Keratoconus match index.

**Figure 5:**
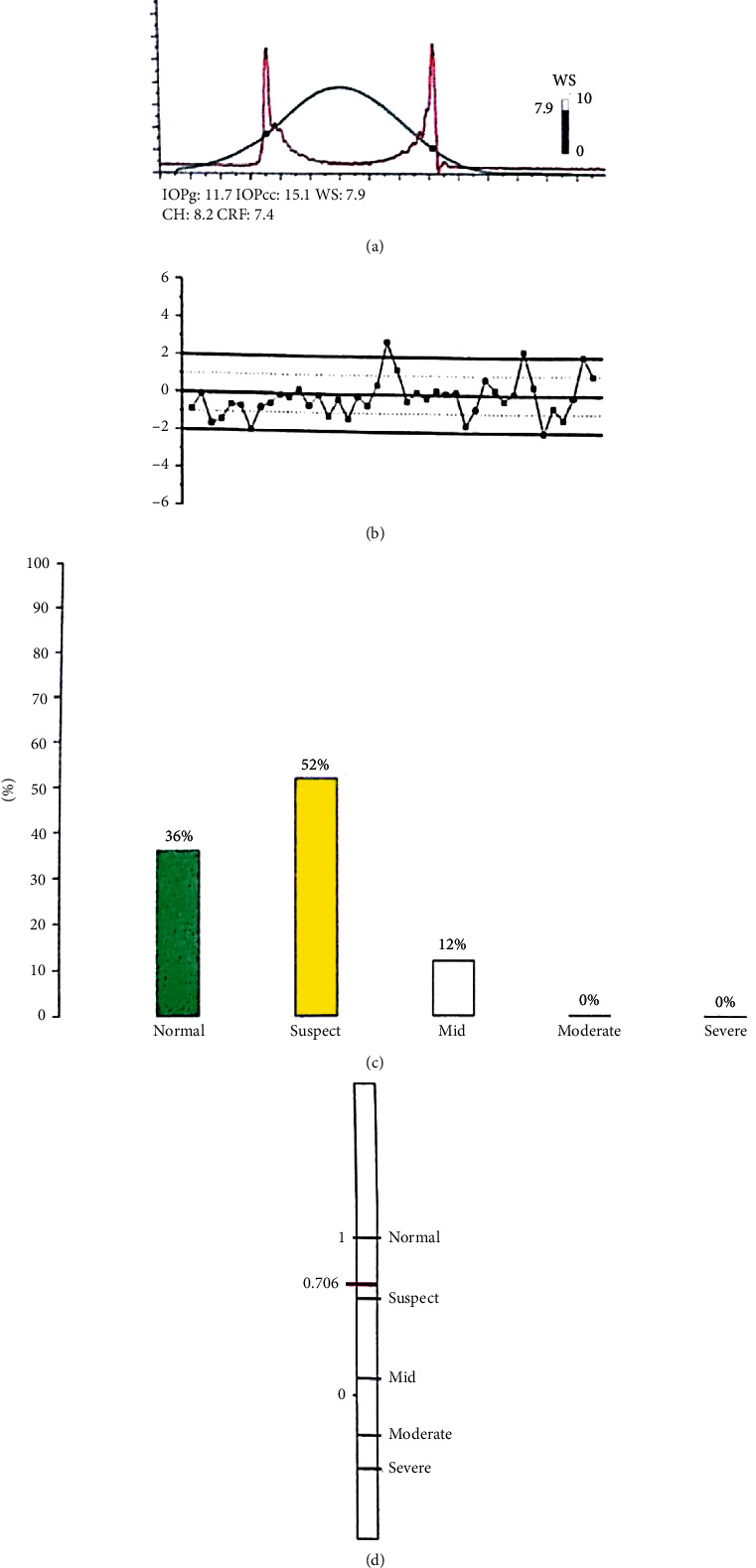
ORA signal of the left eye of a patient in group 2 (keratoconus suspect). (a) Waveform and measurement values. (b) Waveform parameters deviation from mean. (c) Keratoconus match probabilities. (d) Keratoconus match index.

**Figure 6:**
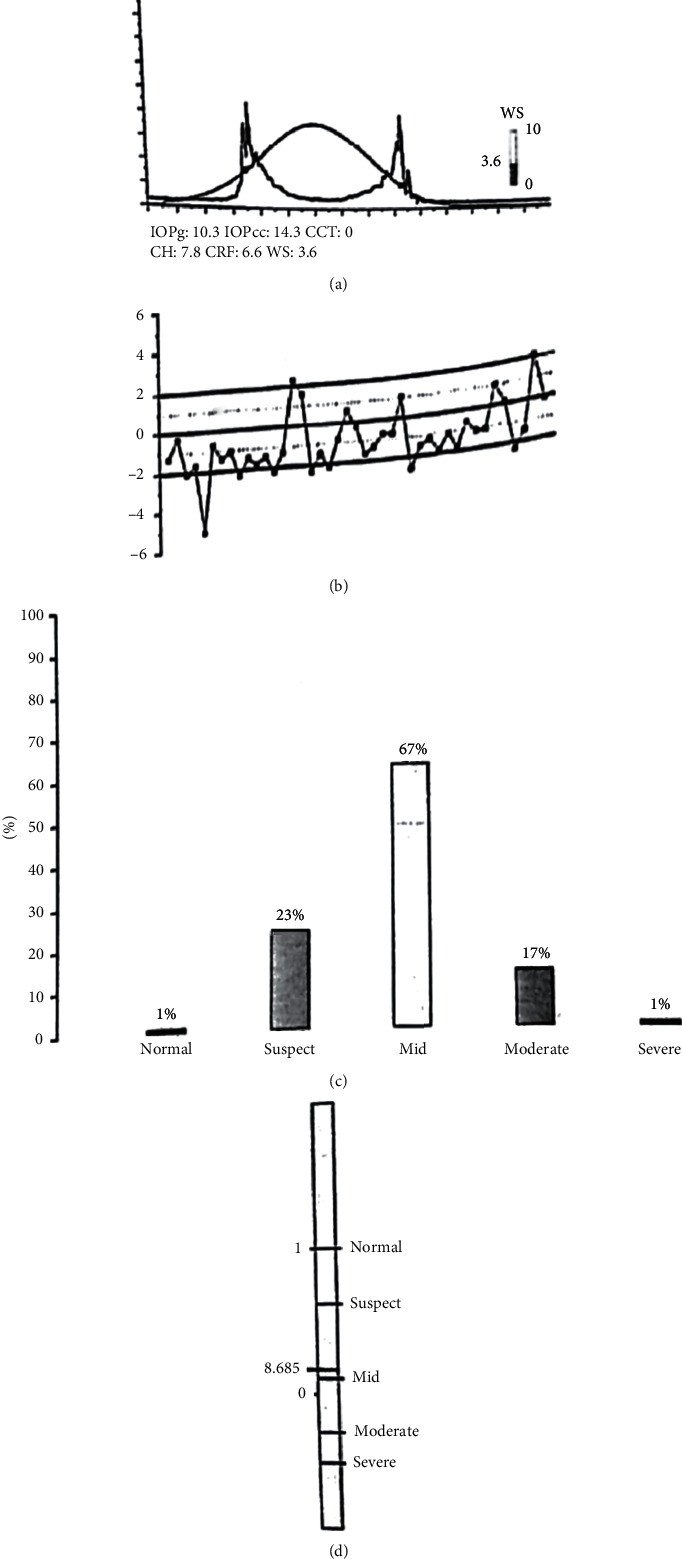
ORA signal of the right eye of a patient in group 3 (keratoconus). (a) Waveform and measurement values. (b) Waveform parameters deviation from mean. (c) Keratoconus match probabilities. (d) Keratoconus match index.

Figures 4–6 represent the ORA signals provided from our three study groups.

The red curve is the “dynamic map” of the cornea obtained during the rapid-in/out deformation. That dynamic process generated two signal peaks that defined the two applanation states. The difference between these inward and outward motion applanation pressures (*P*1 and *P*2) was called corneal hysteresis (CH).

The ORA software utilized the CH to generate two additional parameters: the corneal-compensated IOP (IOPcc) and the corneal resistance factor (CRF). A Goldmann-correlated IOP (IOPg) was also provided by the machine.

## 3. Statistical Analysis

Data were statistically described in terms of mean ± standard deviation (SD), median and range, or frequencies and percentages when appropriate. Comparison of numerical variables between the study groups was done using independent samples *t* test. For comparing categorical data, chi-square (*χ*^2^) test was performed. Fisher's exact test was used instead when the expected frequency is less than 5. Comparison of the continuous variables was done by one-way ANOVA with Bonferroni correction for post hoc analysis. The predictive ability of the ORA parameters was analyzed using receiver operating characteristics (ROC) curve. *P* values less than 0.05 were considered statistically significant. All statistical calculations were done using computer program SPSS (Statistical Package for the Social Science; IBM Corp., NY, USA) version 21 for Microsoft Windows. ROC curves were developed using MedCalc biomedical statistics software version 15.8 (MedCalc Software bvba, Ostend, Belgium).

## 4. Results

A total of 103 eyes from 58 subjects were enrolled in our study, of which 44 eyes showed normal thin (NT) corneas, 26 eyes showed keratoconus suspect (KCS), and 33 eyes showed frank keratoconus (KC).

The age of our study groups ranged from 17 to 37 years, with an average of 27.47 ± 3.02 years for group NT, 25.35 ± 5.42 for group KCS, and 29.37 ± 3.86 for group KC (Table 1).

Our study included 44 eyes from 22 healthy controls (13 males and 9 females), 26 eyes from 16 KCS subjects (10 males and 6 females), and 33 eyes from 20 KC patients (12 males and 8 females). The gender distribution is summarized in Table 2.

The central corneal thickness ranged from 470 to 500 *μ*m, with an average of 490.60 ± 7.07 *μ*m for group NT, 487.64 ± 7.47 *μ*m for group KCS, and 484.31 ± 8.42 *μ*m for group KC. The difference between the three groups was statistically insignificant (*P* value = 0.057) (Table 3).

The mean CH of the study groups was 8.689 ± 1.775, 9.051 ± 1.1190, and 8.129 ± 0.8539 mmHg in NT, KCS, and KC eyes, respectively (Table 3), which is statistically insignificant. The mean CRF of the study groups was 8.441 ± 1.663, 8.337 ± 1.114, and 7.2422 ± 1.3110 mmHg in NT, KCS, and KC eyes, respectively, which was significant only between NT and KC (Table 3).

**Figure 7:**
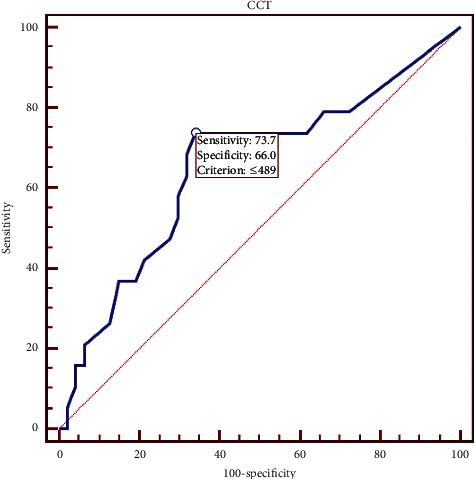
Central corneal thickness (CCT) receiver operating characteristic curve.

**Figure 8:**
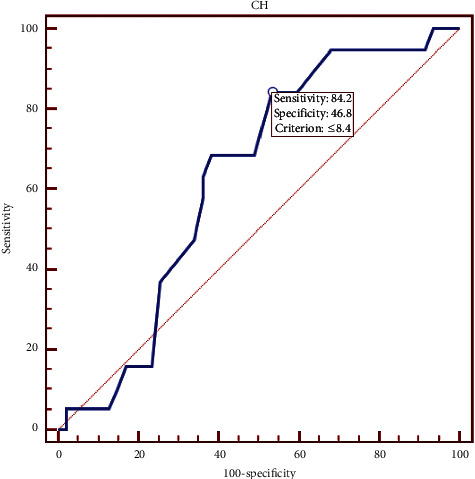
Corneal hysteresis receiver operating characteristic curve data.

**Figure 9:**
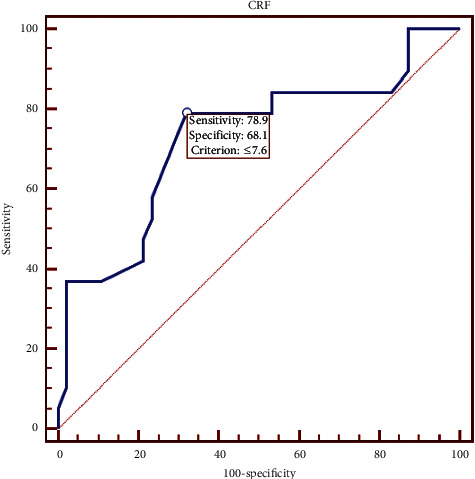
Corneal resistance factor receiver operating characteristic curve data.

The receiver operating characteristic (ROC) curve analysis of central corneal thickness (Figure 7) showed that the optimal cutoff point was 489 *μ*m with 73.68% sensitivity and 65.96% specificity. Also ROC curve analysis of CH showed that the optimal cutoff point was 8.4 mmHg with 84.2% sensitivity and 46.8% specificity (Figure 8) while the optimal cutoff point was 7.6 with 78.95% sensitivity and 68.09% specificity for CRF (Figure 9).

## 5. Discussion

Forme fruste keratoconus (FFKC) and keratoconus suspect (KCS) diagnoses remain a dilemma, despite the advances in using topographic and tomographic tools; there is no specific accepted consensus for categorizing an eye as KCS [10]. Many cases of postrefractive corneal ectasia are still reported, and that is why searching for a supplementary investigation to detect FFKC and KCS is needed. For decision making to perform corneal ablation procedure in these cases, the surgeon should depend on analysis of multiple investigations and parameters [11–15].

Ocular response analyzer (ORA) represents a relatively new perspective for in vivo measurement of corneal biomechanics; since its development by Luce [5], many studies have evaluated the ORA parameters (CH and CRF) for detecting keratoconus (KC) and keratoconus suspect (KCS) and normal thin (NT) eyes [16–20]. Other reports have determined that CH and CRF are significantly lower in KC eyes than in NT eyes and reported CH and CRF as poor properties for discriminating mild KC from NT eyes [6, 21, 22]. In spite of the various studies performed to evaluate the ORA accuracy for detecting KC and KCS from NT eyes, the diagnostic performance of the CH and CRF remains of limited value and the role of CCT as a confounding factor is not yet clearly defined [6, 16–20].

The current study tried to reveal the diagnostic value of ORA as an auxiliary test to differentiate thin corneas with different topographic diagnoses (KC, KCS, and NT). Our results showed that only the mean CRF was significantly lower in KC eyes compared to NT ones, but no significant difference was seen in CCT, CH, and CRF parameters of KCS eyes compared with NT eyes.

Various studies have assessed the CH and CRF between NT and KC eyes. Fontes et al. [6] found significantly lower CH and CRF in KC in comparison to NT eyes. However, we found that only the CRF was significantly lower in KC than NT eyes, with no significance to CH.

Our study shows comparable results to Galletti et al. [21] who prove that corneal resistance factor was better than CH for detecting keratoconic corneas once the effect of CCT on ORA measurements was considered, even for topographically unaffected fellow eyes of patients with keratoconus. The CCT-corrected CRF cutoff values and transformed indices may be of clinical use. In other words, CH is probably decreased in eyes with keratoconus but not to the point that it can be clinically useful in ORA-based subclinical keratoconus detection.

The present study also demonstrated that the mean CH, CRF, and CCT in KCS did not differ from NT eyes. Using the principle Orbscan criterion to identify KCS that was a difference of 1.5 diopters or greater between superior and inferior corneal curvature, we did not find any significant difference between groups. Saad et al. used a computer-based calculation from Nidek OPD scan videokeratographer and found a significant difference between NT and KCS first, which failed to remain significant after controlling for CCT [22].

A possible hypothesis for this finding might be the mysterious role of corneal thickness on corneal biomechanics. CH and CRF are known to be highly correlated to corneal thickness [6, 23, 24]. As corneal thickness decreases significantly in keratoconic eyes [25] and usually is within NT limits in KCS and NT eyes, any changes in CH and CRF could be related to the changes in CCT. After controlling for the CCT in our study, only CRF differences between NT and KC remained significant. The CCT between NT and KCS were not significantly different and therefore could not play a confounding role.

Schweitzer et al. [18], on the contrary, evaluated the performance of the ocular response analyzer (ORA) in the screening of FFKC. They found a significant difference between NT and KCS with the ORA which provided additional information in the screening of FFKC. Furthermore, Johnson et al. [16] studied the difference in corneal biomechanical properties, after controlling for potentially confounding factors, along the spectrum of keratoconic disease as measured by the keratoconus severity; they concluded a significant difference in the mean CH and CRF between normal and FFKC corneas after controlling for differences in age, sex, and central corneal thickness. However, there is a significant overlap in the distribution of CH and CRF values among all groups. The biomechanical parameters CH and CRF cannot be used alone but may be a useful clinical adjunct to other diagnostic tools, such as corneal tomography, in distinguishing normal from subclinical keratoconic corneas. The lack of proper definition or grading for keratoconus suspects leads to discrepancies in the interpretation for different studies handling this subject.

As the receiver operating characteristic (ROC) curve analysis between KC and NT eyes showed, selecting the cutoff points for CH (8.4) and CRF (7.6) provided 84.2% sensitivity and 46.8% specificity for CH and 78.95% sensitivity and 68.09% specificity for CRF. There was no significant difference between KCS and NT eyes in CH and CRF. Mohammadpour et al. [26] showed the ROC curve analysis between KC and NL eyes, which showed that selecting the cutoff points for CH (8.75) and CRF (8.45) provided the predictive values of 84% and 91.4%, respectively. However, Fontes et al. [7] reported a poor overall predictive value of CH (74.83%) and CRF (76.97%) with the cutoff points of 9.64 mmHg and 9.60 mmHg, respectively.

The ORA parameters could be beneficial in differentiating KC eyes from NT ones, but they cannot differentiate KCS from NT eyes. So, the ultimate challenge is to have a test that could discriminate KCS with high sensitivity and specificity from NT eyes.

## 6. Conclusion

CRF only can be helpful in differentiating KC from NT eyes; KCS could not be predicted with either corneal biomechanical metrics. CH has no role in differentiating between the three study groups. The current technology for corneal biomechanical assessment needs further refinement in order to highlight the suspected corneal ectasia early.

## Figures and Tables

**Table 1:** The age of the study groups in years.

	Group 1 (normal thin)	Group 2 (keratoconus suspect)	Group 3 (keratoconus)
Mean ± SD (in years)	27.4738 ± 3.02526	25.3567 ± 5.42305	29.3732 ± 3.85634
Range (in years)	24–35	17–37	18–33
*P* value	0.061		

**Table 2:** Gender distribution in both groups.

	Group 1 (normal thin)	Group 2 (keratoconus suspect)	Group 3 (keratoconus)	Total
Males	13	10	12	35
Females	9	6	8	23
Total	22	16	20	58
The Pearson chi-square	0.806			

**Table 3:** Central corneal thickness (CCT) and the corneal hysteresis (CH) and the corneal resistance factor in three groups.

		Group 1 (normal thin)	Group 2 (keratoconus suspect)	Group 3 (keratoconus)
*CCT*	Mean ± SD (in *μ*m)	490.60325 ± 7.0776848	487.64233 ± 7.470756	484.3142 ± 8.415676
	Range (in *μ*m)	470–500	470–500	470–500
	*P* value	0.059		

*CH*	Mean ± SD (in mmHg)	8.6893 ± 1.7757	9.0512 ± 1.11909	8.1297 ± 0.85395
	Range (in mmHg)	5.9–14.20	7.30–10.80	6.30–10.90
	*P* value	Between groups 1 and 2	0.713	
		Between groups 2 and 3	0.149	
		Between groups 1 and 3	0.711	

*CRF*	Mean ± SD (in mmHg)	8.4413 ± 1.6632	8.3373 ± 1.1144	7.2422 ± 1.3110
	Range (in mmHg)	5.6–12.50	6.70–10.10	5–10.00
	*P* value	Between groups 1 and 2	0.94	
		Between groups 2 and 3	0.075	
		Between groups 1 and 3	0.021	

## Data Availability

The datasets used and/or analyzed during the current study are available from the corresponding author upon reasonable request.

## Abbreviations

NT: Normal thin cornea
KC: Keratoconus
KCS: Keratoconus suspect
CCT: Central corneal thickness
ORA: Ocular response analyzer
CH: Corneal hysteresis
CRF: Corneal resistance factor
IOPg: Goldmann-correlated intraocular pressure
IOPcc: Cornea-compensated IOP
ROC: Receiver operating characteristic.
